# General infection prevention, mitigation, and control procedures implemented in the university education during the COVID-19 pandemic to achieve classroom attendance: a successful community case study

**DOI:** 10.3389/fpubh.2023.1309902

**Published:** 2024-02-21

**Authors:** Diana Monge, Ana Gallego-Gil, Fernando Neria, Soledad Canellas, Fernando Caballero, Ana Díaz de Bustamante, Mónica Samper

**Affiliations:** ^1^Facultad de Medicina, Universidad Francisco de Vitoria, Madrid, Spain; ^2^Servicio de Seguridad, Salud y Bienestar, Universidad Francisco de Vitoria, Madrid, Spain; ^3^Unidad Técnica de Estrategias Poblacionales en Vacuna, Subdirección General de Prevención y Promoción de la Salud, Dirección General de Salud Pública, Madrid, Spain

**Keywords:** health and safety, education, COVID-19, university, prevention, containment measures

## Abstract

**Introduction:**

The COVID-19 pandemic entailed confinement and elimination of face-to-face university classes in Spain. The Francisco de Vitoria University in Madrid (UFV by its Spanish acronym) implemented risk management systems to enable on-campus university activity to avoid a negative impact on students, teachers, and faculties.

**Methods:**

A tracking/registry system was implemented to collect data, identify COVID-19-related cases, implement containment measures, and do follow-up in the UFV community (administration/services personnel [ASP], teaching/research personnel [TRP], and students), from September 2020 to April 2022. In addition, a prevention plan was implemented on campus to avoid COVID-19 spreading. Satisfaction with these measures was assessed through an online questionnaire.

**Results:**

A total of 7,165 suspected COVID-19 cases (84.7% students, 7.7% ASP, 6.5% TRP) were tracked (62.5% female cases, mean age (±SD) 24.8 years (±9.2 years)), and 45% of them confirmed (82% symptomatic/16% asymptomatic), being the student group that with the highest percentage (38.3% total tracked cases). The source of infection was identified in 50.6% of the confirmed cases (90.2% located off-campus). Nineteen COVID-19 outbreaks were registered (inside-10/outside-9). COVID-19 incidence rates were similar or lower than those reported in the Community of Madrid, except in the last wave, corresponding to Omicron variant. The degree of satisfaction (scale 1–6) with the implemented measures was high (scores 4.48–5.44).

**Conclusion:**

During the COVID-19 pandemic, UFV control measures, periodic monitoring, and the effectiveness of the tracking system have contributed to maintaining classroom teaching, guaranteeing health and safety. UFV has adapted to a new reality as an example of good practice for future pandemics or emergency situations.

## Introduction

The coronavirus disease 2019 (COVID-19) was declared a pandemic on 11 March 2020 by the World Health Organization (WHO). It hit hard worldwide, and Spain was one of the most affected European countries (1, 2), with its capital, Madrid, the epicenter of COVID-19 (2).

The health crisis caused by COVID-19 led to an adaptation of society that has been an unprecedented challenge (3). The spreading severe acute respiratory syndrome coronavirus 2 (SARS-CoV-2) resulted in a state of emergency in Spain in March 2020 that involved a confinement as an urgent measure to contain the virus infections (4). The availability of vaccines and doses led to a vaccine strategy developed in three phases, starting with an initial and very limited supply of vaccine doses (first stage) and increasing doses and vaccines to cover all priority groups (third stage) (5). The Spanish vaccine campaign started in December 2020, prioritizing the population most vulnerable to serious disease (residents and staff in nursing homes and centers for older adults and care of major dependents; front-line health and social-health personnel; other health and social-health personnel; non-institutionalized major dependents) (5). From February to June 2021 (second stage), the following groups were vaccinated: older than 80; people between 70 and 79 and people with very high-risk conditions; people between 60 and 65; persons between 66 and 69; other health and social-health personnel; workers with an essential social function; people between 50 and 59. The third stage, starting June 2021, covered the remaining population between 49 and 5 years. In this last stage, vaccination sites were opened in up to 26 public and private universities in Madrid, to facilitate access to vaccination and achieve better coverage.

As in other regions, the state of emergency in Spain entailed that face-to-face classes were eliminated from March to September 2020 (6), leading to the introduction of new educational and administrative solutions in university management (7). Universities had to reorganize their activities with immediacy and creativity to avoid a negative outcome for students’ education (4). They had to adapt quickly their campuses to online teaching quickly to continue with their activities, without sufficient training and with a high level of improvisation (8).

It has been reported that remote learning generated many problems for students, teachers, and faculties, even in highly developed countries (7, 8). Students generally took the virtual transition negatively, with behavioral and emotional changes that affected their wellbeing and academic performance (4, 9). The negative impact of repeated closures of educational institutions has demonstrated that this strategy should be a last resort, and reopening them should be considered a priority (7).

After the March 2020 confinement in Spain, the 2020–2021 academic year started with the distance education or blended mode in public universities and most private ones.

The action plans recommended at the university level by the authorities resulted in distance university teaching with a low face-to-face content (10). However, Francisco de Vitoria University (UFV), a private university located in Madrid, implemented risk management systems, making it possible to continue face-to-face education by establishing a rotation system in classroom attendance.

As in other universities (11), UFV established several control measures to control COVID-19 transmission on the campuses. The impact of COVID-19 on the UFV community has been studied using a proprietary tracking system and demonstrating that with appropriate prevention and containment measures, university campuses are safe. Herein, we describe the preventive measures, case detection, and containment systems carried out on campus since the beginning of the COVID-19 pandemic to maintain on-campus university activity with the necessary teaching quality for the students, as an example of good practice for future pandemics or emergency situations. As a secondary objective, we also described the satisfaction of the UFV community with the measures implemented.

## Methods

### Study design

An observational study was conducted between September 2020 and April 2022 to describe the COVID-19 cases reported in the UFV university.

### Settings and participants

Data collected for the analysis included the information on cases reported in the UFV community. This community is composed of administration and services personnel (ASP), teaching and research personnel (TRP), and students. The study period includes from the beginning of the academic year 2020–2021 (September 2020) to the change of strategy in the follow-up of cases and the end of the mask in indoor spaces, April 2022. During this period, data from the second to the sixth wave (Omicron variant wave) of the COVID-19 pandemic have been recorded.

### Variables, data sources, and measurements

During this study, a tracking and registry system was established in which a group of variables were measured. The following sociodemographic and epidemiological variables were collected for case detection and follow-up: age, sex, group (student/TRP/ASP), faculty, case type (confirm case/close contact/suspect), vaccination status, possible source of infection, date of symptom onset, and last date of attendance to the campus.

#### Definitions and data sources

**Figure 1:**
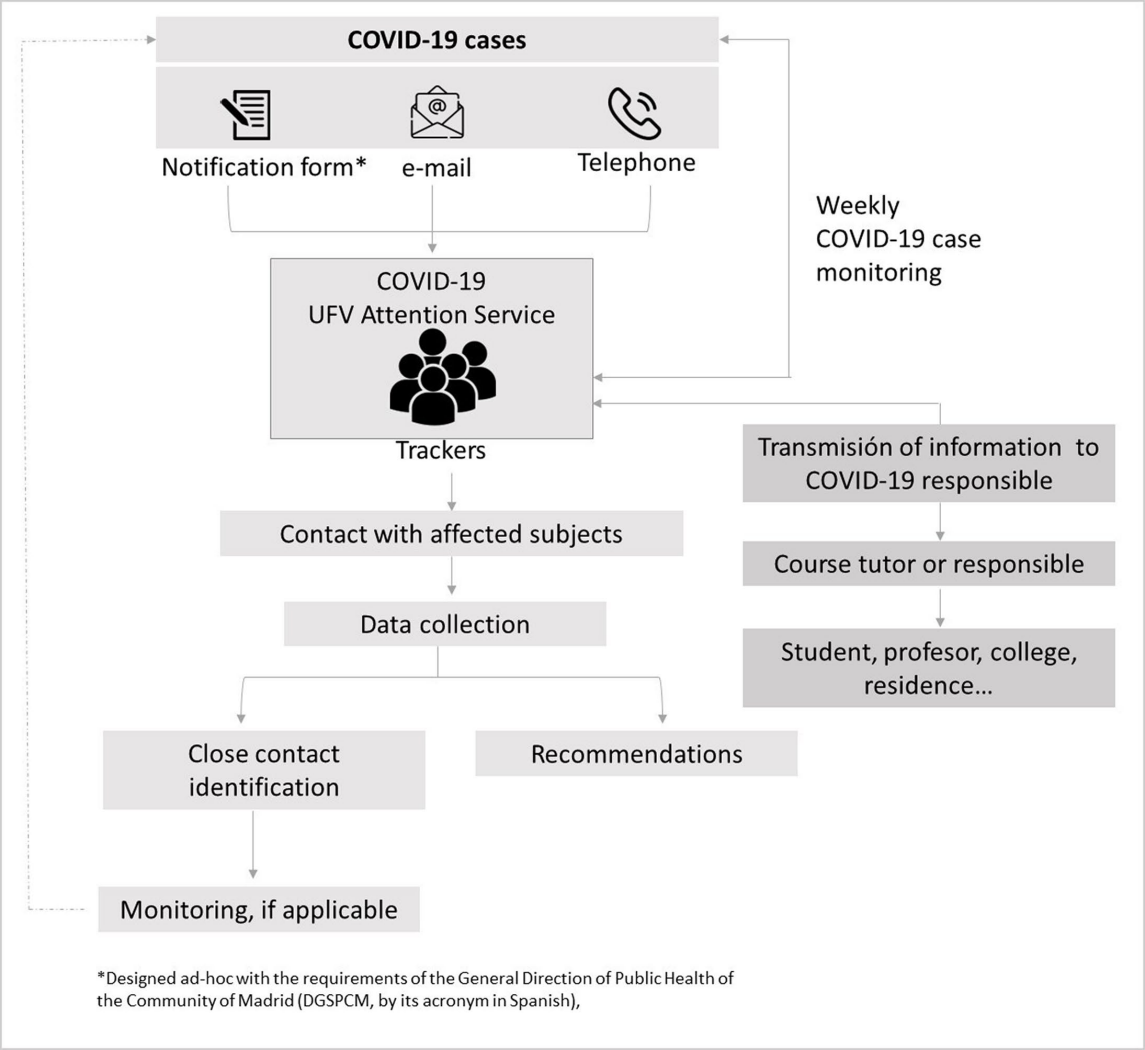
Flowchart of the COVID-19 case registration system used to identify, classify, and follow up cases in the UFV during the pandemic.

The *tracking/registry system* implemented by UFV from the beginning of the 2020–2021 academic year, according to indications of the Spanish Health Authorities, is shown in Figure 1.

This system enabled the identification, classification, and monitoring of COVID-19 cases for rapid action and adequate containment of virus transmission on the UFV campus/community. It enabled the analysis of the epidemiological link between cases for rapid decision-making on campus, considering if appropriated/recommended immediate containment measures such as the switch of in-person classes to remote or facilitating teleworking until the transmission risk situation was considered to have been resolved.

A suspected COVID-19 case (12) or person with compatible symptoms (13) was defined as any person with a clinical condition of acute respiratory infection of sudden onset of any seriousness presenting cough, dyspnea, throat pain, or rhinorrhea, with or without fever. Other symptoms, such as odynophagia, anosmia, ageusia, muscle pain, diarrhea, chest pain, or headache, may also be considered symptoms of suspected SARS-CoV-2 infection according to clinical criteria.

A confirmed COVID-19 case with active infection (12) was defined as a person who meets clinical criteria for a suspected case with a positive Diagnostic Test for Active Infection (DTAI) COVID-19, an asymptomatic person with a positive DTAI, and people with positive results in antigen self-testing dispensed in pharmacies in epidemiological situations of high incidence (14).

The trackers who received the alerts of COVID-19 infections contacted the affected subjects to collect data (possible origin of the contagion, date of symptom onset, last date of attendance to the campus, places, and activities carried out in previous days, etc.), identified and classified close contacts, and, if necessary, followed them up (Figure 1).

A close contact (12) is defined as any person who has had contact with the case from 2 days before the onset of symptoms (or the date of sample collection for diagnosis in the case of asymptomatic subjects) until the time the case is isolated, and (A) has provided care to a case or has been in contact with its secretions and fluids: health or social-health personnel who have not used appropriate protective measures, or persons who have other similar physical contact or any person who has handled biological samples without the appropriate protective measures; and/or (B) has been in the same place as a case, at less than 2 meters and for a total accumulated time of more than 15 min in 24 h. In environments where an assessment of the prevention measures could be made, including the correct and continued use of the mask, an individualized assessment was made by the occupational risk prevention service.

The above definitions were modified over the months according to the appearance of new diagnostic tests, the vaccination status of the population, and increased epidemiological evidence and knowledge regarding the virus (new strains, transmissibility, etc.).

According to each case, trackers explained the situation, provided the recommendations to be followed (e.g., if the identified close contacts were on campus, they were instructed to leave the facilities and were referred to their primary healthcare center for proper control and monitoring of their health status) and provided guidance on the quarantine and lockout periods indicated by the health authorities.

COVID-19 outbreak was defined as any grouping of three or more cases with active infection in which an epidemiological link has been established (12). If an outbreak was identified, it was reported to health authorities.

When the possibility of an outbreak was identified (>2 cases and/or a high number of close contacts), health authorities were informed, and the group leader was contacted and the whole class changed temporarily (between 2 and 5 days) to remote learning. After monitoring the cases and contacts, the group was gradually allowed to return to face-to-face training depending on their level of exposure (from lowest to highest risk of infection).

Trackers monitored the evolution of the cases weekly and updated the registries, including any change of condition.

All information provided through an electronic questionnaire was recorded; possible new cases were followed up by repeating the process in Figure 1.

The source of infection was established in two ways: by follow-up of registered close contacts who subsequently became confirmed cases or by a telephone survey asking whether they had had close contact with a confirmed case in the 10 days prior to the symptoms or positive result.

#### Measures for the prevention of infection

The UFV health and safety action plan included the implementation of measures on staff organization at the university, and infection prevention (protection and hygiene material, cleaning, disinfection, and ventilation), and also, the dissemination and awareness of habits to prevent the spread of COVID-19. The measures carried out to promote and guarantee a safe campus, where students and the rest of the community could continue with on-campus university activities, are shown in Table 1.

**Table 1:** Measures considered in the UFV to promote and guarantee a safe campus during the COVID-19 pandemic.

**Measures of hygiene**
Hydroalcoholic gel pumps at the entrance of all classrooms, in customer service areas, and in common areas.
Waste containers for removal of masks and gloves.
Daily reinforcement of cleaning and disinfection throughout the campus, paying special attention to public areas, classrooms and laboratories, toilets, and offices, as well as unique spaces for events. Emergency cleaning and disinfection if cases were detected on campus.
Increased ventilation levels: continuous switching on of air fresheners, natural ventilation whenever possible, switching on of all toilet exhaust fans 24/7, and reinforced cleaning of the ventilation system.
**Information and training measures**
Safety protocols for the different university activities and an awareness campaign aimed at the entire university community: training sessions, distribution of posters, infographics, and videos, and open access to a specific web site.
Periodic dissemination of best practices aimed at the entire university community (more intensively during exam periods), including access to lectures and presentations of interest about the COVID-19 pandemic.
Awareness campaign for the entire university community, especially students, to encourage individual responsibility and solidarity, including the signing of a responsible declaration for safe coexistence on campus.
“If you take care of yourself, you take care of me” guidelines provided to UFV students and employees with basic health and safety measures to be followed on campus.
Awareness campaign “Live Christmas in a special but safe way” aimed at the entire university community to avoid contagions during these dates.
**Organizational and technical measures related to the activity**
An emergency committee, as a decision-making entity, which adopts decisions upon identification of transmission sources in the University community.
Expansion of the surveillance services team for greater control of compliance with COVID-19 regulations on campus to ensure safe coexistence.
A capacity control system in enclosed spaces with a large influx of visitors.
Signage on campus to regulate the flow of movement of the UFV community (access, routes, distances…).
Construction of new spacious and well-ventilated common areas for eating, studying, etc.
Massive videoconferencing systems in the classroom to be able to integrate students connected from home remotely.
Reduction of the size of the in-person groups for the master classes while preserving the distance between students.
Master classes adapted to a mixed teaching (remote from home and in-person in the classroom) alternating so that students maintain the highest teaching quality and preserve their academic activity.
Small groups of students for in-person practical’s lecturers in laboratories and other spaces (clinical/surgical simulation center, sports center, film sets and recording area, etc.), and increase the number of practical’s lecturers to achieve practical training for 100% of the students.
**Management measurements**
A centralized service to respond to and accompany all COVID-19 members of the university community, which attends and follows them up and tracks close contacts.
A COVID-19 UFV manager who is responsible for the COVID-19 people in charge of each faculty center and the trackers.
A COVID-19 case management system for an adequate control of the evolution of the pandemic on campus. Continuous reporting system of COVID-19 cases, data dump, and exploitation of pandemic evolution reports at the UFV.
Weekly follow-up meetings in the Emergency Committee and with the COVID-19 managers to evaluate the evolution of the infections and make quick decisions.
Smoking ban on campus (October 2020) to avoid unprotected facial exposure.
Mandatory use of face masks in indoor areas.
UFV provided FFP2 masks to the campus staff (in second wave) and asked students to use them.

Key: FFP2: Filtering face pieces 2; UFV (by its Spanish acronym): Francisco de Vitoria University.

Additionally, in the third phase of the vaccine strategy, when vaccines were available (2020–2021 and 2021–2022 academic years), the UFV promoted the vaccination through communications to students. A vaccination campaign was specifically promoted on the UFV campus, from 11 h to 18 h, in September (days 20–22) and October (days 13–14) 2021.

When infections increased, non-essential administration and services personnel (ASP) and professors were asked to work from home.

#### Satisfaction measurement

Satisfaction with the measures implemented during the 2020–2021 and 2021–2022 academic years, at the UFV campus, for COVID-19 prevention/ management/ containment and promotion of healthy living at UFV was assessed using three items (1. “*The university promotes a healthy lifestyle inside and outside the work environment*,” 2. “*The university raises awareness of COVID-19 in the university community,”* 3. “*The support provided by the university to the university community during the pandemic is adequate*”). They were included in a non-validated quality questionnaire done by the Quality and Institutional Assessment Department of the UFV. These satisfaction items were designed by the Safety, Health, and Welfare Service of the UFV. Each item was rated using a six-point Likert scale from 1 (lowest degree of agreement) to 6 (highest degree of agreement). Data collection was performed online for 15 days.

The questionnaire was distributed to all first-, third-, and fifth-year students of all the degrees taught at the university and to ASP and teaching and research personnel (TRP).

### Study size

Since this is a descriptive study of the evolution of COVID-19 cases reported at the university throughout the study period, the entire university population (students, TRP, and ASP) was included.

### Statistical methods

Qualitative variables were described as absolute (*n*) and relative frequencies (%). Quantitative variables were described by mean and standard deviation. The cumulative incidence of COVID-19 cases diagnosed in periods of 14 days in the UFV was calculated by dividing the number of new cases by the number of people who are free of the disease at the beginning of the period. To compare cumulative incidence between populations, a paired *t*-test was performed. Statistical analysis was performed with the software R v4.2.3.

### Ethics statement

This study on the effect of prevention, identification, and management systems implemented at the UFV during the COVID-19 pandemic was approved by the UFV Institutional Review Board (IRB).

## Results

### Tracked subjects

**Figure 2:**
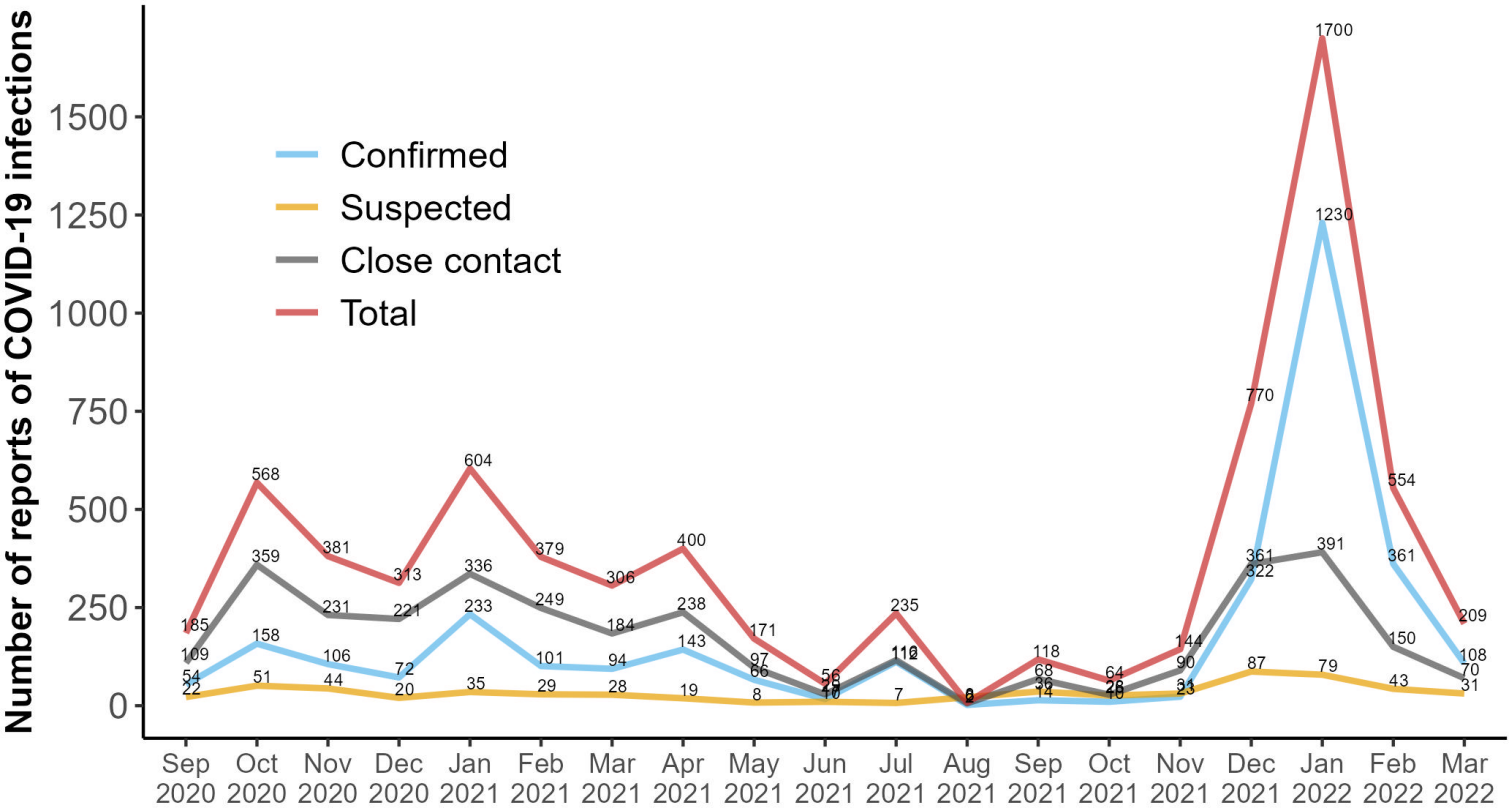
Frequency of monthly reports related to COVID-19 during the collection data period recorded by the tracking system (7,165 total cases). Cases classified as confirmed, suspected and close contacts are shown. A fourth line representing the total number of cases (corresponding to the sum of the three previous categories) is also presented.

From September 2020 to April 2022, 7,165 cases (62.5% female cases) were tracked in five (second to sixth) COVID-19 waves (Figure 2).

Overall, the mean age (±SD) of the subjects was 24.8 years (±9.2 years): students, 21.6 (± 3.3) years; ASP, 43.0 (± 10.3) years; and TRP 44.7 (± 9.5) years.

The most tracked subjects were UFV students, 6,070 (84.7%) subjects, followed by 555 (7.7%) ASP, 463 (6.5%) TRP, and others (101, 1.1%). By faculty, those with the most cases tracked were Health Sciences (30.5%), Legal Business (16.6%), and Communication (15.1%), which coincide with those with the largest number of students. Table 2 shows this distribution stratified by teaching area within the University, including the type of tracked cases.

**Table 2:** Types of tracked cases for TRP/ASP and students by UFV schools or faculties (*n* = 7,165).

	Type of case *n*, (%)			
	Confirmed	Suspected	Close contact	Total
**TRP**	220 (6.8%)	34 (5.6%)	209 (6.8%)	463 (6.5%)
**ASP**	204 (6.3%)	44 (7.3%)	307 (9.2%)	555 (7.8%)
**Other**	59 (1.8%)	1 (0.2)	41 (1.2%)	101 (1.1%)
**Students**				6,047
Postgraduate	86 (2.7%)	21 (3.5%)	71 (2.1%)	178 (2.5%)
Faculty/school				
Center for Technological and Social Studies (CETYS)	140 (4.3%)	24 (4.0%)	159 (4.8%)	323 (4.5%)
Higher Polytechnic School	220 (6.8%)	62 (10.2%)	194 (5.8%)	476 (6.6%)
Communication Faculty	451 (14.0%)	77 (12.7%)	550 (16.5%)	1,079 (15%)
Experimental Science Faculty	305 (9.5%)	35 (5.8%)	277 (8.3%)	617 (8.6%)
Legal Business Faculty	576 (17.9%)	86 (14.2%)	525 (15.8%)	1,187 (16.6%)
Health Sciences Faculties				2,187 (30.5%)
Physical activity and sports sciences, nursing, physiotherapy, and nutrition	478 (14.8%)	117 (19.3%)	492 (14.8%)	1,087 (15.2%)
Education and Psychology Faculty	283 (8.8%)	71 (11.7%)	288 (8.6%)	642 (9.0%)
Medicine Faculty	204 (6.3%)	34 (5.6%)	220 (6.6%)	458 (6.4%)
Total, *n*	3,226 (45.0%)	606 (8.5%)	3,333 (46.5%)	7,165 (100.0%)

ASP: administration and services personnel; CETYS: Center for Technological and Social Studies; TRP: Teaching and Research Personnel; UFV (by its Spanish acronym): Francisco de Vitoria University.

Overall, 45% (3,226) of the tracked cases were confirmed (2,637 [82%] cases had COVID-19 symptoms, and 501 cases [16%] remained asymptomatic; the remaining 88 patients did not report data about symptoms), and 46.5% (3,333) remained as close contacts who did not test positive (Table 2).

The student group was the group with the highest number of confirmed cases (2,743/3,226; 38.3% of total tracked cases).

### Causes of origin of infection in confirmed cases

The source of infection was identified in half of the confirmed cases (1,632, 50.6%). Most 90.2% (1,472) had an off-campus source of infection (Supplementary Figure S1A), while 160 cases were linked to on-campus activity (Supplementary Figure S1B). In each of these setting (off- and on-campus, respectively), the most common source was partners (49.5%) and meals on campus (36.9%).

### COVID-19 outbreaks

Nineteen COVID-19 outbreaks were registered, both identified inside (*n* = 10) and outside (*n* = 9) the campus (parties, outside celebrations, or travel) with a total of 337 affected subjects: 159 positive cases and 178 close contacts that did not become positive. The mean number of cases per COVID-19 outbreak was 8.4 subjects (4.8 and 12.3 subjects in outbreaks identified inside or outside the campus, respectively), and the mean of affected cases (confirmed cases + close contact) was 17.7 (11.4 and 24.8 subjects in outbreaks identified inside or outside the campus, respectively). The mean number of close contacts that become positive per case was 6.3 (4.5 and 9.6 in outbreaks identified inside or outside the campus, respectively).

Only in four COVID-19 outbreaks, there were confirmed cases that were a close contact of the initial case. In three of these outbreaks, the number of confirmed cases was elevated. Their origin (big parties and/or travel) made it difficult to identify the contacts. However, it helped health authorities to identify early COVID-19 outbreaks in these places.

### Evolution of the epidemiological curve

**Figure 3:**
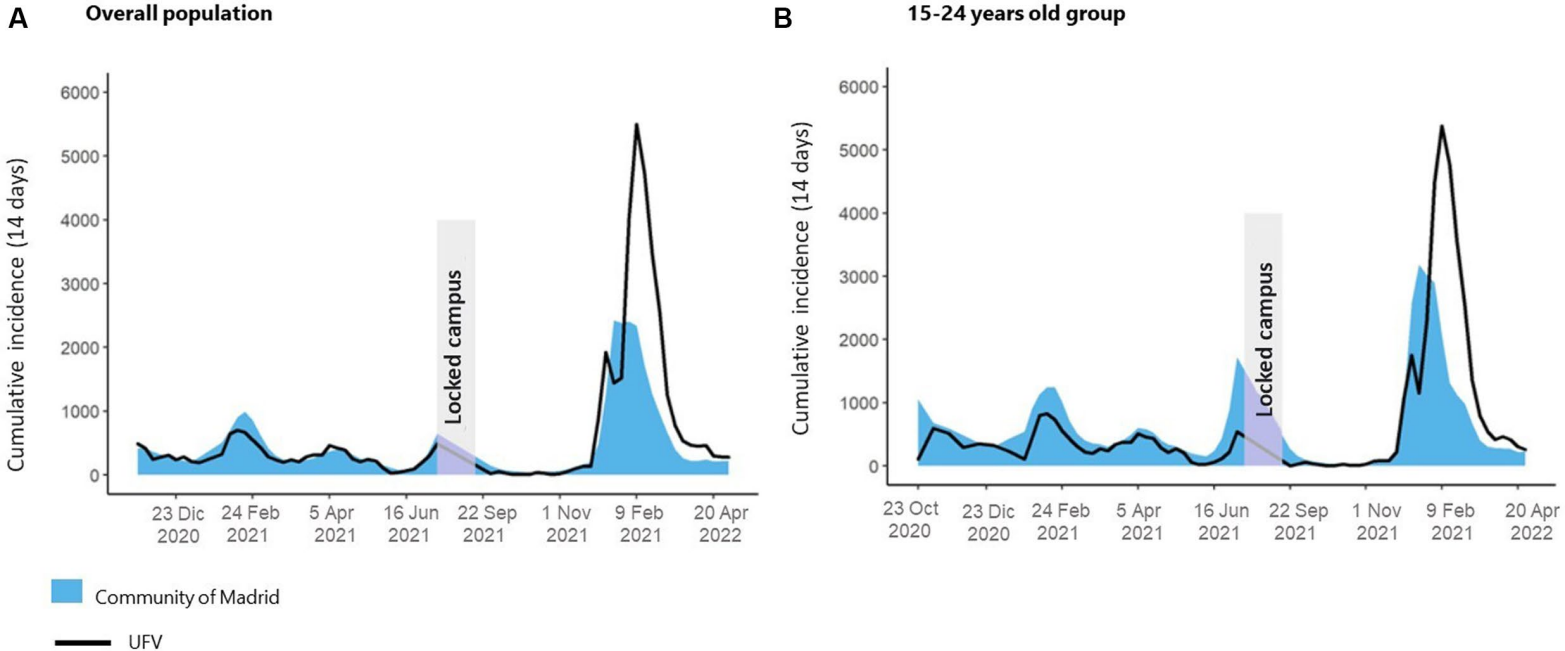
Comparison between COVID-19 incidence rates at the UFV (black line) vs. Community of Madrid (blue area) in the overall population **(A)** and 15-24 years old group **(B)** from October 2020 to April 2022. Second to sixth wave (Omicron variant) are included in this period.

The cumulative incidence of COVID-19 cases diagnosed in periods of 14 days in the UFV, from September 2020 to April 2022, is shown in Figure 3. It was compared with the incidence reported by the Community of Madrid (CM), both in the overall population (Figure 3A) and in the 15–24 years old group (because most of our university population are students with a mean age of 21.6 years [85.4%; 2,754 of the 3,226 confirmed cases]) (Figure 3B).

Despite the on-campus activity, in both populations assessed, COVID-19 incidence rates reported in UFV were similar or lower than the ones reported in the Community of Madrid most of the time, except in the last wave registered, corresponding to Omicron variant. No significant difference was found between cumulative incidence in both populations (15–24 years *p*-value: 0.830; overall *p*-value: 0.068, paired *t*-test).

### COVID-19 tests

After the identification of confirmed cases and the later study of close contact and suspect cases, 6,059 (84.6%) out of the 7,165 tracked cases had reported that they used a diagnostic test (DTAI): 36.7% antigen test, 36.8% antigen self-test sold in pharmacies, and 26.5% PCR.

Confirmed cases are defined as those with a positive DTAI; hence, 100% of the confirmed cases had a diagnostic test performed (with a positive result). In total, 87.8% of the suspect and 71% of close contacts cases had performed DTAI. If a suspect or a close contact case tested positive during the follow-up, they were re-identified as a confirmed case.

### Vaccination

Since the vaccination register started (March 2021), a total of 4,387 confirmed positives were recorded; 734 of them belonged to the population that, at the time of infection, did not yet have access to the vaccine. Of the remaining confirmed cases within the UFV community that could have been vaccinated at the time of infection, only 304 (8.3%) of those cases notified did not have even one dose of the vaccine, although they belong to an age group with access to it.

During the vaccination campaign on the UFV campus (September and October 2021), 396 first doses and 235 second doses were administrated.

### Satisfaction

The support provided by the university during the pandemic and the measures implemented in the UFV campus were evaluated with a questionnaire as a secondary objective of this study. The participation rates during 2020–2021 and 2021–2022 academic years, respectively, were 56.9% (341) and 41.0% (346) for ASP, 46.9% (551) and 35.3% (449) for TRP, and 30.0% (1,328) and 32.8% (1,708) for students. The results are shown in Supplementary Table S1.

## Discussion

Universities are centers for welcoming and creating communities where students learn and acquire the necessary skills to develop in the next professional stage of their lives.

During the university stage, it is necessary to guarantee not only the acquisition of knowledge but also an experience of personal and relational development that contributes to the maturation process of each student. Without this campus experience, the student may feel isolated and would not complete his or her learning cycle, which is so necessary at this stage of life.

Several studies carried out in universities (15, 16) and other educational centers (schools and high schools) (17–19) agree with our results on the effectiveness of a layered mitigation approach for reducing infections. In fact, it has been reported that layering multiple interventions could reduce infection rates by 75% (20).

This approach includes the use of masks, information and awareness, contact tracing, and measures of confinement (COVID-19 cases and contacts). In addition, as we observed, the application of control measures maintained the educational centers with contagion levels similar to the areas where they were located (Community of Madrid in our study), avoiding, to a great extent, high level of contagion within the campuses. We corroborate that, using appropriate preventive measures, the teaching centers do not necessarily amplify virus transmission but rather reflect the transmission level in the community (21). During a period when most universities experienced low attendance rates (10), we successfully upheld teaching activities on the campus. This accomplishment was attributed to our meticulous adherence to preventive measures and the implementation of a robust case tracking system, ensuring both adequacy and effectiveness. In fact, COVID-19 incidence rates reported in the UFV were similar to or lower than those reported in the Community of Madrid in all waves, except the Omicron one. The more transmissible Omicron variant could justify the increased incidence of the latter, which triggered many infections (22). Moreover, in this wave, most of the confirmed cases were diagnosed using self-testing of antigens purchased in pharmacies, which in many cases were not registered by the health authorities.

Our study population, with a mean age of 24.8 years, showed behavioral risks; in fact, most of the identified sources of infection came from outside the campus (mainly social gatherings and partners). Similarly, in another university also with face-to-face teaching since August 2020 and similar measures as these implemented in the UFV, the source of infection was mostly outside the campus (18). Moreover, the COVID-19 outbreaks we identified in parties or travels showed many more people affected than those identified inside the campus.

Several studies carried out in university showed that the source of infection was high in the first courses classes (20, 23) (age of students approximately between 18 and 19 years). In our campus, the mean age of our student population (21.6 years) suggests a higher proportion of students in courses beyond the first academic year.

Although smoking was the source of infection in few confirmed university-related COVID-19 cases (*n* = 8), the UFV decided to ban smoking after the first wave registered in October 2020. This measure aimed to prevent physical approximation and salivary fluid contact that makes it a risk factor for COVID-19 (24). Currently, smoking is still banned because it also involves a risk of other infectious agents acquired from the environment through respiration (24).

Compliance with the measures is fundamental to control virus transmission on the campuses. It must be considered that this compliance can vary depending on the moment, on the alerts communicated by the health authorities and, in short, on the perception of risk (11). A lower perception of COVID-19 severity and health responsibility in students (the youngest people) vs. university staff and in non-medical faculties has been described (11). We can only highlight a higher accumulation of tracked cases in those faculties with a larger number of students (Health Sciences, Legal Business, and Communication faculties), regardless of whether they are related to health.

The early identification of suspected cases is also essential to interrupt the spread of the virus (6, 25), especially asymptomatic spread (25). The early detection of cases and close contacts prevented the on-campus activity of these subjects and, therefore, avoided new close contacts and new cases when close contacts became positive. Therefore, we show our system for communication strategies and contact tracing. Similar models have already shown success (25), allowing the early identification and isolation of suspected cases and rapid implementation of infection control that entails a rapid decrease in infected cases (26).

Ongoing surveillance, including serosurveillance, plays a critical role in monitoring infection and transmission of SARS-CoV-2 in educational settings (6). On our campus, the method to confirm positive cases was DTAI, performed in the Community of Madrid health centers as established in the Community’s health protocols of the first waves. These protocols varied over the months, and, eventually, during the Omicron wave (January–February 2022), the use of antigen self-tests sold in pharmacies became generalized. Testing for COVID-19 was not always indicated or available. For this reason, only 6,059 (84.6%) cases among the 7,165 tracked cases were tested (DTAI).

In Madrid, the highest number of doses administered of COVID-19 vaccines was recorded between June and October 2021; UFV supported this coverage by the administration of 396 first doses and 235 s doses administrated at the campus (September–October 2021). Since March 2021, the 91.7% of the confirmed cases had, at least, one dose of the vaccine.

University measures, including mitigation efforts and vaccination, may facilitate resumption of normal campus operation (16) and can avoid the negative impact of remote learning reported during the COVID-19 pandemic (4, 7–9). The UFV experience has shown to be effective in maintaining the propagation of cases, with good acceptance among all groups present on campus over the time (2020–2021 and 2021–2022 academic years). The degree of satisfaction was high, with scores ranging from 4.48 to 5.44 on a scale from 1 to 6. The TRP was the group with the highest scores followed by ASP and students. The fact that TRP is the group exposed the most to potential contagions is a possible reason that might explain the maximum scores obtained in this population.

Considering the psychological distress among university students and the relevant lack of motivation for distance learning (9) observed in the COVID-19 pandemic, we showed useful measures to safety reach face-to-face learning and avoid the virtual transition in an emergency situation. In future emergencies, the evidence of COVID-19 infections, including our successful measures and tracking system, could help to promote more specific surveillance strategies. The dissemination of all findings regarding prevention of spreading virus is relevant to be able to act accordingly and improve the COVID-19 or potential future pandemic.

## Limitations

Although the prevalence data could be underestimated due to the diagnostic techniques purchased in the pharmacy and, therefore, not recorded by official sources, this information was available at the university through interviews with the trackers.

Indeed, as this was an observational study, causality cannot be concluded, but the data show incidence rates throughout the study period that were lower than the average for the Community of Madrid, in spite of maintaining a high face-to-face learning.

## Conclusion

In conclusion, the UFV control measures during this pandemic, its periodic monitoring to maintain continuous improvement and the effectiveness of the tracking system implemented, have contributed to maintaining classroom teaching at the UFV, guaranteeing the health and safety of those on campus. This enables the highest teaching quality for students without detriment to the scrupulous compliance with COVID-19 health standards.

Keeping campuses alive, with face-to-face activity, is essential for student and a hallmark for many universities, and during the pandemic, this institution has adapted to a new reality by ensuring quality teaching and research on campuses that have proven to be safe.

## Data availability statement

The raw data supporting the conclusions of this article will be made available by the authors, without undue reservation.

## Ethics statement

The studies involving humans were approved by UFV Institutional Review Board. The studies were conducted in accordance with the local legislation and institutional requirements. The ethics committee/institutional review board waived the requirement of written informed consent for participation from the participants or the participants’ legal guardians/next of kin because the data were previously anonymized by external personnel before being analyzed by the research team.

## Author contributions

DM: Conceptualization, Data curation, Formal analysis, Investigation, Methodology, Project administration, Resources, Validation, Visualization, Writing – original draft. AG-G: Conceptualization, Data curation, Formal analysis, Software, Validation, Visualization, Writing – original draft. FN: Data curation, Formal analysis, Investigation, Methodology, Validation, Visualization, Writing – original draft. SC: Visualization, Writing – review & editing, Investigation, Validation. FC: Visualization, Writing – review & editing, Conceptualization, Funding acquisition, Resources. AD: Investigation, Validation, Visualization, Data curation, Software, Writing – review & editing. MS: Conceptualization, Investigation, Project administration, Supervision, Validation, Visualization, Writing – original draft.
