# Supplementary material for: General infection prevention, mitigation, and control procedures implemented in the university education during the COVID-19 pandemic to achieve classroom attendance: a successful community case study
Source: Front Public Health. 2024 Feb 21;11:1309902. doi: 10.3389/fpubh.2023.1309902 (PMC10915036; doi:10.3389/fpubh.2023.1309902)
Supplement: Supplementary file 1 [file Table_1.docx]

**Supplementary Table 1.** Degree of agreement with the items related to the COVID-19 measures implemented at the UFV campus during 2020-21 and 2021-22 academic years.

|  | **Degree of agreement with the item**  **Mean scores (1-6-scale)** | | | | | |
| --- | --- | --- | --- | --- | --- | --- |
| Item | **ASP** | | **TRP** | | **Students**  **(1^st^-, 3^rd^- 5^th^- year)** | |
|  | 2020-  2021 | 2021-  2022 | 2020-  2021 | 2021-  2022 | 2020-  2021 | 2021-  2022 |
| The university promotes a healthy lifestyle inside and outside the work environment | 5 | 4.8 | 5.1 | 5.1 | 4.7 | 4.6 |
| The university raises awareness of COVID in the university community | 5.2 | 5.1 | 5.4 | 5.5 | 4.9 | 4.7 |
| The support provided by the university to the university community during the pandemic is adequate | 5.2 | 5.0 | 5.3 | 5.3 | 4.6 | 4.5 |

Key: ASP: administration and services personnel; TRP: Teaching and Research Personnel; UFV (by its Spanish acronym): Francisco de Vitoria University
