# Supplementary figures and images for: General infection prevention, mitigation, and control procedures implemented in the university education during the COVID-19 pandemic to achieve classroom attendance: a successful community case study
Source: Front Public Health. 2024 Feb 21;11:1309902. doi: 10.3389/fpubh.2023.1309902 (PMC10915036; doi:10.3389/fpubh.2023.1309902)

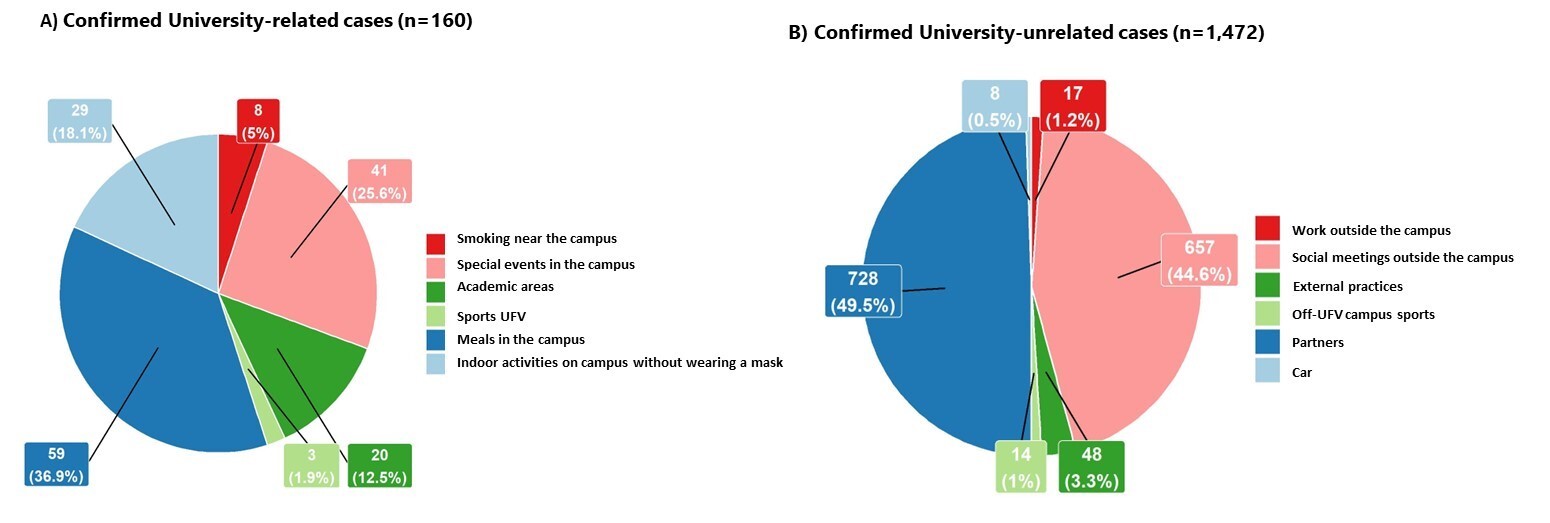

Supplement: Supplementary file 2 [file Image_1.JPEG]
